# Norwegian district medical officers’ experiences during Covid-19: A qualitative study

**DOI:** 10.1177/14034948251394900

**Published:** 2025-12-02

**Authors:** Bettina C. Fossberg, Jan C. Frich, Ivan Spehar

**Affiliations:** Institute of Health and Society, University of Oslo, Norway

**Keywords:** Crisis management, district medical doctors, Covid-19, pandemics, qualitative research, healthcare systems, public health administration, community health services, community medicine

## Abstract

*Background/aims:* Municipalities in Norway are required to have a district medical officer (DMO) as chief medical advisor and leader of infection control and prevention work. There is great variation between municipalities in how the role is enacted and organized. Before the Covid-19 pandemic many DMOs felt their role to be unclear and invisible in the organization. This study aimed to explore DMOs’ experiences during the Covid-19 pandemic and how organizational context mediated these experiences. *Methods:* We conducted a qualitative study, generating data from three focus groups involving 17 DMOs from three different regions in Norway. Data were analysed using systematic text condensation. *Results:* All DMOs, regardless of municipal size, position size or infection rate, reported feeling overwhelmed by the responsibility during the pandemic. Their organizational outset, number of tasks, number of enquiries and uncertain foundation for decisions contributed to this experience. Organizational characteristics that promoted DMOs’ coping were resource allocation, teamwork, and professional and social support. *Conclusions:* Our results suggest that municipalities can enhance their crisis management of future infection control crises by establishing an organizational structure around DMOs that facilitates networking, and by developing plans for task delegation and creation of teams to support and alleviate DMOs during a crisis.

## Background

In Norway, the primary responsibility for organizing infection control and prevention lies with municipalities and the district medical officer (DMO) [1]. Each municipality is mandated by law to employ a DMO as a medical advisor [2] but can decide on the administrative arrangements. DMOs are medical professionals specializing in community medicine, a field that encompasses public health, health preparedness, environmental health protection, health leadership, and infection control and prevention. The role acts as local health authority through legislation within most of these fields.

There are three levels of authorities in the Norwegian health system: national level, regional level and municipal level. National authorities provide national legislation and advice, regional authorities follow up on national regulation and coordinate within the region. Municipal authorities give health services in line with the national legislation and regional advice – but can establish local regulations that are more stringent than national, if considered necessary [3]. In a crisis, the Norwegian management rests on four principles: responsibility, similarity, proximity and cooperation. In short, these principles state that a crisis should be managed at the lowest possible level similar to normal circumstances [3]. This gives the municipal leaders authority and responsibility to manage a crisis locally, in collaboration with regional and national authorities.

Different theoretical models of crisis management have been developed. In 2007 Coombs presented a three-staged approach [4], inspired by earlier models by Fink [5] and by Mitroff [6]. The three phases all had distinctive features and overlapped each other in a circular matter. The pre-crisis phase represents normal circumstances where warning signs can occur, and organizations should prepare and reduce risk for crisis. The crisis event phase unfolds an unstable situation with need for rapid decisions. Managing involves recognizing the crisis, controlling it and communicating. In the post-crisis phase the crisis resolves and stability is restored. Through learning and revising the organization can move on to the pre-crisis phase again, better prepared [4]. Several studies on pandemic management support and align with this overarching theoretical model, elaborating on different roles of crisis management leaders [7] and the importance of communication and teamwork [7–10].

The Covid-19 pandemic represented a crisis for the municipal infection control management. The infection hit Norway’s central areas around big cities harder than rural areas [11]. As infection control leaders, DMOs in different municipalities faced different infection rates. But there were also considerable differences between municipalities regarding the organizational placement and scope of the DMO role [3,12]. A qualitative study by Fossberg and Frich in 2019 found that just before the pandemic hit Norway, many DMOs felt their role was poorly defined and experienced being invisible in their organization [13]. However, during the Covid-19 pandemic the DMOs were key in managing the local infection control and prevention work. A Norwegian Official Report that evaluated the pandemic response concluded that the DMOs were crucial for managing the pandemic but had lacked the necessary prerequisites for handling the situation and therefore had faced a demanding work situation [3].

In light of the varied organizational context of the DMO role, the DMOs’ experiences before the pandemic and the large scope of the pandemic, we aspired to gain knowledge about the DMO role during the Covid-19 pandemic to facilitate for improved crisis management in future infection control crisis.

### Aim

The aim of this study was to explore the experiences of DMOs during the Covid-19 pandemic, particularly in relation to how the organizational context influenced these experiences.

## Methods

### Design

We found that a qualitative design was suitable for exploring DMOs’ experiences [14], using focus group interviews to generate data. Such interviews might facilitate interaction between participants that can bring forth experiences and reflections that might not emerge in individual interviews [14,15]. The Standard for Reporting Qualitative Research (SRQR) checklist was used in the writing process [16].

### Participants and sampling

Participants in our study were DMOs who had worked during February 2020 to December 2022. We used a purposive sample strategy and recruited a maximum-variation sample aiming at diversity in local infection rate, geography and municipal population size. Two local DMO networks were contacted in different regions with large municipalities and high infection rate during the pandemic. DMOs who met the specified criteria within these groups were invited by email to participate in the study. A third focus group consisted of participants from a region with less populated municipalities and low infection rate during the pandemic (Table I).

**Table I.** Description of the focus groups in the study.

Region	Group	Regional infection rate during the pandemic	DMOs contacted (met the criteria)	DMOs recruited	Municipalities represented
Region A	Natural group	High	9	7	4
Region B	Natural group	High	7	5	4
Region C	Focus group	Low	32^a^	5	6

a Not known how many of these met the criteria for participation.

DMO: district medical officer

We were unaware of an existing DMO network in this area but contacted the county governor, who forwarded an invitation to 32 DMOs in the region. The number of DMOs that met the criteria is unknown, but five DMOs were recruited, one of whom worked across two municipalities, thereby representing a total of six different municipalities.

In total, we conducted three focus groups with 17 DMOs representing 14 different municipalities in three different regions in Norway. Our sample varied in gender, age, experience, academic rank/competence, and contracted working hours (Table II). The participants defined the overall infection rate of their own municipality compared with the national average after the following criteria:

**Table II.** Description of the study population.

Participant number	Age	Gender: male (M) or female (F)	Contracted working hours in % of a full position	Municipal population at the start of pandemic 2020 (SSB)	Experience as district medical officer at the start of the pandemic	Specialist in community medicine	Overall infection rate during the pandemic
1	36	F	100	20,000–29,999	< 5 years	No	High
2	64	F	100	⩾ 100,000	> 10 years	Yes	High
3	63	M	100	90,000–99,999	5–10 years	Yes	High
4	61	F	100	90,000–99,999	5–10 years	No	High
5	43	M	40	⩾ 100,000	5–10 years	No	High
6	41	M	100	⩾ 100,000	< 5 years	No	High
7	69	M	100	⩾ 100,000	> 10 years	Yes	High
8	40	F	100	80,000–89,999	5–10 years	Yes	High
9	44	F	100	80,000–89,999	5–10 years	Yes	High
10	67	F	40	40,000–49,999	> 10 years	Yes	Medium
11	49	M	100	40,000–49,999	5–10 years	Yes	High
12	49	F	100	30,000–39,999	> 10 years	Yes	High
13	41	M	50	0–9999	> 10 years	No	Low
14	62	M	50	20,000–29,999	> 10 years	Yes	Medium
15^a^	50	F	60	10,000–19,999	5–10 years	Yes	Low
15^a^	50	F	40	0–9999	< 5 years	Yes	Low
16	50	M	40	0–9999	< 5 years	No	Low
17	67	F	60	10,000–19,999	5–10 years	Yes	Medium

a Participant 15 worked in two different municipalities and is therefore listed in two rows.

Municipal population data were obtained from Statistics Norway (Statistisk sentralbyrå, SSB).

- High: high infection rate with constant outbreaks throughout the period;
- Medium: average infection rate, some periods with outbreaks and/or higher infection rate;
- Low: low infection rate, few periods of outbreaks and/or higher infection rate.

Using this method 50% of the municipalities in the study were defined to have a high overall infection rate during the pandemic period, 21% a medium infection rate and 29% a low infection rate (Table II).

Participation in the study was voluntary. Recruitment through self-selection inherently carries a risk of bias, as it remains uncertain whether individuals who chose not to participate have different experiences compared with those who did. Self-selection may also result in the inclusion of participants with more pronounced opinions compared with those not participating. In this study, the topic addressed was one that most DMOs were eager to discuss. To promote inclusion, we conducted the group interviews at a location selected by the participants. Using natural groups, we aimed to establish a safe environment for sharing diverse experiences, recognizing that group interviews can sometimes inhibit the expression of divergent or marginal viewpoints [15]. To mitigate potential negative group dynamics, researcher BCF acted as a mediator during the interviews, ensuring that all participants had an opportunity to voice their perspectives in the group discussion.

### Data collection and analysis

The three focus group interviews were conducted during February and March 2023. The interviews lasted on average 80 min, using a semi-structured interview guide that was developed by the research group. Reflections that occurred during the interviews were explored by the moderator (BCF) and followed up with further probing questions. All interviews were audio recorded and manually transcribed by BCF and read through by IS. Participants were anonymized in accordance with current privacy regulations and ethical approval. The audio recordings were deleted.

The data were analysed using Systematic Text Condensation [17] in four steps: gaining an overall impression of the data, identifying and sorting meaningful data units into code groups, dividing code groups into subgroups and condensing meaningful units within each subgroup into a short text, and synthesizing the condensed texts into analytic texts representing each code group – making overarching themes. To ensure validity and representativeness, BCF and IS read through the transcripts to make sure the final analytic texts gave a true impression of the original material. Special attention was given to statements that contradicted the analytic findings. All authors contributed to the analysis. Crisis management literature was used as a theoretical framework, but analytic categories evolved from the empirical data and were not made in advance.

### Reflexivity

First author BCF is a district medical officer, with first-hand experiences related to the research theme. To avoid affirming prejudice, several measures were made. First, BCF wrote a log revealing thoughts and experiences on the theme before and during the research process. This was repeatedly looked back at to enhance reflexivity [18–20]. There was extensive contact between BCF and IS throughout the research process, to discuss findings and challenges, and clarify thoughts and expectations [17]. Themes and results were then discussed with JCF, who also contributed to the writing process. IS and JCF are researchers at the University of Oslo, with a social science and medical background respectively, and not connected to the DMO field.

### Ethics

The study has been approved by the Norwegian Centre for Research Data (ref. no: 782023). All participants received written information about the study before the interviews and signed a consent form.

## Results

Our main finding was that the legal responsibility of organizing infection control and prevention work felt overwhelming during the pandemic for all DMOs in the study, regardless of municipal size or local infection rate. The feeling of being overwhelmed especially occurred when the situation surpassed what they felt they could manage, and appeared hard to overcome. The organizational context could both mediate this experience and relieve it. We identified two overarching themes: ‘Increasing the burden to bear’ and ‘Organizational relief of DMOs’.

### Increasing the burden to bear

In our study, DMOs described four main factors that added to the burden of responsibility to the extent that it became overwhelming. These were: poor organizational outset, number of tasks, number of enquiries, and uncertain foundation.

### Organizational outset gave different starting points for DMOs

The DMOs in our study operated within different organizational contexts when the pandemic hit. Few participants described an organizational context that facilitated their work as infection control leaders at the start of the pandemic. Most participants had 100% positions, but some had part-time positions combined with general practitioner work, and lack of resources for infection control work was a common theme. Part-time positions proved to be less flexible and difficult to combine with all the work during the pandemic. Few described having local infection control arenas in the municipality where the overall and systematic work on infection control was done.

We found that a lack of organizational support could be offset by well-functioning networks. Participants with broad relations before the pandemic described easier access to support and resources when needed and, because of that, less feeling of being overwhelmed. Experienced DMOs with small networks described the same struggle to get support as DMOs with short experience and few relations.

‘I feel that before the pandemic I was invisible, and there were many in the management of the municipality who did not know that I even existed.’ (Focus group 3)

They gave various reasons for weak relations, including organizational characteristics like placement at the bottom of the hierarchy, small position size, municipal mergers and physical location outside the town hall. The feeling of little visibility in the organization was particularly raised by DMOs from medium-sized and large municipalities. Poor organizational outset exacerbated DMOs’ challenge in obtaining relief during the pandemic as their workload increased, resulting in a greater sense of isolation regarding the responsibility. This contributed to their feeling of being overwhelmed.

### An endless number of tasks

Participants described an overwhelming number of tasks that had to be dealt with, owing to the scale of the pandemic and lack of fitting plans. The tasks were associated with infection control work and included organizing contact tracing, public Covid-19 testing, managing the quarantine of close contacts and isolation of Covid-19 positive individuals, and facilitating public vaccination. Additional tasks encompassed administrative work with recruitment and development of local regulations. These new tasks were added to the responsibilities DMOs already had in other domains. The large number of tasks led to a feeling of being stretched, working on many different administrative levels in the municipality at once.

‘I find it strange that in a crisis, the person who does the individual follow-up of patients is the same person that keeps in touch with national directorates, handles the media and the overall crisis management. I had to be superior but also work right down to the details. It was a huge contrast and an insane workload’ (Focus group 2)

Participants described workdays of 15–18 h with no weekends off and difficulty sleeping at night. Several participants experienced a lack of control over their own time, as they had to manage tasks continuously. Many DMOs experienced little protection of their own health and working environment.

### Flooded by enquiries

The need for communication was experienced as overwhelming by all the DMOs in our study, irrespective of region, municipality size and infection rate. It encompassed responding to enquiries regarding regulations, local infection status and potential risks from the municipal organization, businesses, the public and the media. It also entailed releasing information about new regulations and procedures to the same recipients. Participants described a great need for translating national regulations and advice before communicating them locally. Several said they had spent more time on communication than on actual infection prevention work during the pandemic.

‘One thing that was unexpected, was the tidal wave of enquiries! From all angles. And the need for interpretation, clarification, guidance, and advice both high and low in society.’ (Focus group 2)

There were some differences in the type of communication that was emphasized. Participants from municipalities with little infection were more concerned with getting preventive information out, while participants from municipalities with high level of infection stressed incoming enquiries and the media. All the participants emphasized the importance of their local knowledge to tailor measures and advice to the local conditions and communicate this to the public. Their experience was that local management and communication during the pandemic created ownership, belonging and support in the population, which in turn increased compliance.

### Major decisions based on uncertain foundations

Many participants felt a great personal responsibility for the municipal handling of the pandemic. Some described a surrealistic shift from an almost invisible role in the organization to becoming a figurehead, dictating decisions and controlling the municipal response.

‘From being a somewhat unknown person who walked around doing as I pleased, it was suddenly like. . . NOW . . now we have a district medical officer! I had no resistance in the Crisis management leadership group. They did just about everything I said. The politicians, too.’ (Focus group 1)

National recommendations often shifted and entailed a need for constant changes in the local decisions. The constant pressure for decisions combined with professional uncertainty added to the DMOs’ feeling of being overwhelmed by responsibility. Participants described strong pressure from the educational sector for decisions to close schools, even when this was not professionally recommended. Some also had pressure from politicians, especially in times of high infection rates. Many participants felt alone in the decision-making process. Several described a sense of duty to manage everything themselves, therefore waiting too long before asking for help – and asking for too little. The great workload and responsibility affected them both professionally and personally.

‘There are many people who think that doctors are almost invincible. But I had to take sick leave. It was simply too much for me. I couldn’t sleep, it was a constant buzz in my head. I got awkward at home, and it just didn’t work. So, I want to convey that we are just ordinary people. There is a limit to what we can tolerate.’ (Focus group 1)

In our study, the majority of DMOs described the weight of the local responsibility in a situation characterized by the need for swift decisions based on uncertain professional grounds, with potentially serious consequences for society, life and health. The more isolated they felt within the organization, and the more uncertain the professional basis was, the heavier they perceived the burden of responsibility.

### Organizational relief of DMOs

Through our analysis we identified four organizational factors that seemed to decrease DMOs’ feelings of being overwhelmed: resource allocation, teamwork, professional support and social support.

### Reducing the burden through resource allocation

Participants reported experiencing relief when delegating tasks to other personnel within the municipality. At the onset of the pandemic, they managed contact tracing independently and interacted directly with affected individuals. Eventually, all municipalities in our study allocated resources to establish infection tracking teams, which alleviated some of the responsibilities for DMOs. Additionally, some DMOs received assistance in responding to enquiries, and all noted the formation of vaccination teams. Another approach to alleviate workloads involved digitalization of the contact tracing process, which had initially been conducted manually.

‘It was a great relief when we got the digital infection tracking program in place and an infection tracking team. A heavy burden fell off my shoulders.’ (Focus group 3)

While there were variations in the timing of when and to what degree DMOs were relieved of tasks, all reported some relief from operational infection control responsibilities. Conversely, organizational support for administrative tasks, such as document filing, legal assessments and staff recruitment, was perceived by many DMOs as more challenging to attain. Some were left with the responsibility to allocate staff themselves.

### Teamwork improved crisis management

Teamwork was described by all the DMOs as essential for better crisis management. By sharing responsibility, relieving tasks and supporting decisions teams lessened the burden of responsibility that many DMOs felt overwhelming. All but one participant were part of the municipal crisis management group with the municipal leaders. Most DMOs recounted this team as a good arena for strategic decisions about the overall crisis management and felt recognized as a valuable advisor in the group. Most participants still pointed out a need for additional teams to coordinate and effectuate the operative infection control work.

‘The crisis management group was quickly assembled, and I was part of it. It worked well, but I felt they didn’t quite grasp the practical challenges. So, we established another team, primarily me and the director of health, and we pulled in the people we needed to solve the tasks at hand.’ (Focus group 3)

Common features of successful operative teams were broadly composed and scalable sizes and with participants with authority to make decisions but closeness to the executive field. These teams enhanced the operative crisis management and contributed to relieving DMOs of both tasks and responsibility as decisions were supported by the team. The DMOs who had experienced such operative teams all described them as important success factors during the pandemic.

### Professional support essential in times of uncertainty

The Covid-19 pandemic constituted a period of professional uncertainty owing to the novelty of the virus. The ongoing mutations of the virus led to constant changes in the infection situation, and a significant need for rapid decisions. Within this dynamic context, the DMOs in our study emphasized the critical role of professional support in facilitating improved decision-making. DMOs often serve as the sole doctor within their field in the municipal organization. As a result, professional support was primarily obtained externally from DMO colleagues in nearby municipalities and professionals at the Norwegian Institute of Public Health (NIPH).

All the participants had joined regional groups with DMO colleagues during the pandemic for professional support. Some participants had pre-established networks, others created a network in their region during the pandemic. None of the DMO networks were formalized in local planning or regulations. The need for support from colleagues across municipalities seemed to be present regardless of municipal size, infection rate or number of colleagues locally. The networks provided an arena for professional discussions on complex and demanding matters and opportunity to coordinate decisions and understanding in the region.

‘The professional meeting point, and social and collegial meeting point, was *so* important for me! It enabled me to do a far better job than I would have done without it, because you get to level your decisions properly. It’s so nice to have the collegial community. Not all of us have colleagues where we work.’ (Focus group 2)

The support from professionals at the NIPH was also highlighted in our study, especially by DMOs from large municipalities with high infection rate. They were more often out of step with national advice and experienced pressure for decisions. Participants described the dialogue and professional support from NIPH as crucial as it enabled them to make better decisions and relieved their feeling of being alone with the responsibility. NIPH also helped DMOs gain influence for difficult and unpopular decisions locally.

### Social support enabled DMOs to cope

Our analysis indicates that in addition to professional support, social and mental support was vital for DMOs coping with the situation. Support could come from a local leader, local operative teams, the public or from DMO colleagues. All our participants described the regional DMO network as the most important arena for social and mental support, as they shared similar experiences.

‘Just getting that mental support! It’s like, no one else knows how you feel. Not even the ones at home! Not your husband, not your kids, not your partner. No one understands how you feel. Nobody gets it but this group!’ (Focus group 2)

Participants from smaller municipalities especially emphasized local support from leaders, colleagues, or the public in their municipality. Some participants from large municipalities described little local support and a feeling of being alone. They spoke of the importance of the external arenas for support.

## Discussion

**Figure 1.**
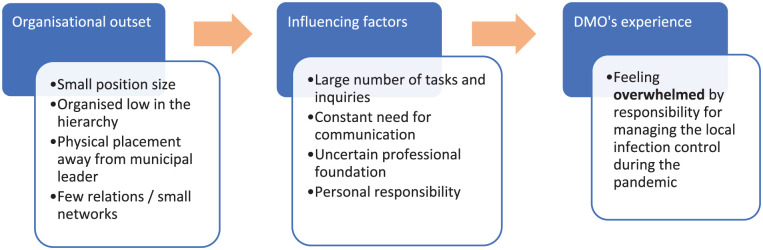
How poor organizational outset and influencing factors during the pandemic led to district medical officers (DMOs) in our study feeling overwhelmed by responsibility.

Our main finding was that DMOs experienced a feeling of being overwhelmed during the Covid-19 pandemic. Studies show that crisis management leaders need to have time and energy to be calm, reflective and able to make good decisions. A leader’s emotional regulation is important for trust, endurance and team functioning in managing complex crisis situations [7,9]. Many DMOs did not operate within an organizational context or had a network that facilitated effective crisis management. Their starting point affected their access to resources and support and, because of that, their experience in the pandemic period. During the pandemic they were flooded with infection control tasks and a need for communication and had uncertain professional grounds to make decisions. They felt a great personal responsibility for managing the situation. These factors increased the experience of being overwhelmed. The results are illustrated in Figure 1.

We identified four factors that reduced this feeling, which were allocation of resources, access to strategic and operative teams, professional support and social support.

If we merge our findings into Coombs’ theoretical model of crisis management [4], we find that the pre-crisis phase in Coombs’ model is equal to our organizational outset. Here we found that few DMOs had organizational structures or plans that promoted access to support pre-pandemic. Factors such as experience, position-size, placement in the organizational structure, physical location and access to arenas for decision all seemed to influence this. This is supported by other studies [11,21,22]. Well-functioning networks seemed to facilitate support as the pandemic hit. Previous research has shown the same [24,25]. Gittell et al. point out that enhancing relations between persons collaborating can increase both quality and efficiency performance [25].

In the crisis event phase, the unstable situation needs to be handled. In our study we found that DMOs felt overwhelmed by the crisis management. Some participants cried during the interviews, and many told of breakdowns during the pandemic. A surprising result was that the experience of being overwhelmed was reported by all DMOs, regardless of municipal size, infection rate or position size. Through the analysis we found that the main reason for lack of difference was the need for communication and the feeling of responsibility. Even though some municipalities had a low infection rate, the need for translating national advice and giving information and guidance was high, and the responsibility felt personal and overwhelming.

The great need for communication is well documented in studies on crisis management [7,8,24]. Crisis communication can help maintain motivation and drive amongst the employees and contribute to trust and compliance in the public [7,8]. Marsen emphasizes that professionals have an important role in crisis communication, as they legitimize the message and ensure trust [8]. Many DMOs in our study also pointed out their experience of local communication as vital for ensuring compliance and trust in the municipality. Studies from crisis management supports this [8,24].

Organizational factors that decreased the experience of being overwhelmed, and by doing that increased crisis management, were administrative relief, access to strategic and operational teams, and professional and social support. Studies show that crisis management inflicts new tasks on leaders and requires quick responses, so the need for administrative support increases [7,9]. We found that many DMOs felt that support for administrative tasks came too late, if at all. In managing a crisis, it is vital for a leader to have time and support to be able to make decisions and communicate [7–9]. The use of teamwork is well documented in crisis management literature as a successful strategy [8,9,24]. In our study most DMOs experienced the municipal crisis management group as a good team for strategic crisis handling, but they still needed additional operational teams to coordinate the operative management. Operational teams with flexibility, diversity and closeness to the executive field were the most successful. This aligns with other studies [9,24].

The importance of support became evident in our analysis. Many of the DMOs felt alone with the responsibility for managing the pandemic locally. They had to make decisions that were unpopular with little research basis and described a need for both professional and social support. This need for support in decision-making was also found in a study on ethical challenges for DMOs [26]. We found that support could be provided within the municipality by teams, leaders, municipal colleagues or the public. In addition, all DMOs described a need for external support. DMOs were often alone in their professional field locally, so they generally sought professional support externally.

**Figure 2.**
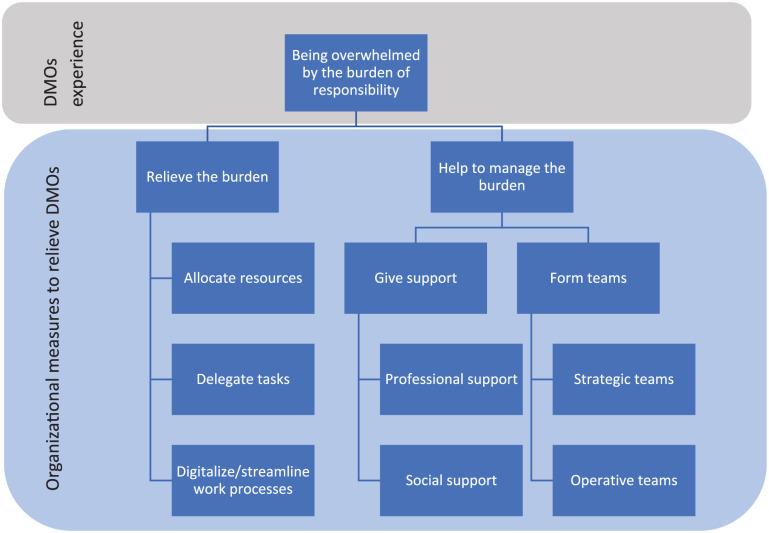
Relationship between district medical officers’ (DMOs’) experience and the municipal measures that were experienced to give relief in our study.

Two external arenas for support stood out as important in our study. The regional networks with DMO colleagues provided support and cooperation, a place of psychological safety. Psychological safety is essential for effective teamwork and learning [27,28], and other studies have also identified colleague networks as important for DMOs during the pandemic [21,26]. The need for regional cooperation during regional and national crises is supported by the literature [9,10]. The DMOs in our study emphasized the importance of professional support from the NIPH. Their advice and professional support helped DMOs make well informed decisions and not give in to local pressure. This aligns with other studies [9,29]. In Figure 2 we illustrate how the different organizational measures were experienced to relieve DMOs’ feeling of being overwhelmed.

In the post-crisis phase, a well-functioning organization will learn from the crisis experience and revise to be better prepared to handle a new crisis. There have been some studies on DMOs after the pandemic, and national evaluation reports have been made [3,11,21–23,26]. But whether the municipalities have learned and revised their crisis management is still unclear. Some studies suggest that there has been little learning and revision, and DMOs are back in a poor organizational frame [22]. Other studies suggest that DMOs are better prepared, but mostly because of relational networks – not organizational structures [23]. In our study we did not go further into the issue of the post-crisis phase, but we believe it is an important field that needs further research.

## Strength and weaknesses

We recruited 17 DMOs with high relevance to the study by selective sampling. Some selection bias cannot be excluded, as participation was by choice. The selective sampling achieved a variation in geography, municipal size, infection rate, experience, position size, age and gender. This added to the validity of the study. The interviews were performed in March 2023. By using focus groups we sought to reduce the effect of recall bias, as the participants engaged in discussions that trigged their memories.

There was some discrepancy between DMOs’ experiences in large municipalities with a high infection rate and in small municipalities with a low infection rate. But the general feeling of being overwhelmed was shared. While the DMO role is distinctive in Norway, the results on factors that influenced and enhanced crisis management might be transferred to similar roles in other places.

## Conclusions

Our results suggest that in significant infection control crises, the DMO role is susceptible to being overwhelmed by the responsibility for local crisis management. Municipalities can enhance crisis management by providing support and task relief to DMOs. We recommend that municipalities assess their organizational structure to promote relationships around DMOs throughout the organization in a pre-crisis phase. Furthermore, municipalities should develop plans for resource allocation and establishment of strategic and operative teams supporting the DMO role during a crisis. A formalization of regional DMO networks in planning and contractual documents is also recommended, to ensure their existence and significance in future crises.
